# The Genome of *Akkermansia muciniphila*, a Dedicated Intestinal Mucin Degrader, and Its Use in Exploring Intestinal Metagenomes

**DOI:** 10.1371/journal.pone.0016876

**Published:** 2011-03-03

**Authors:** Mark W. J. van Passel, Ravi Kant, Erwin G. Zoetendal, Caroline M. Plugge, Muriel Derrien, Stephanie A. Malfatti, Patrick S. G. Chain, Tanja Woyke, Airi Palva, Willem M. de Vos, Hauke Smidt

**Affiliations:** 1 Laboratory of Microbiology, Wageningen University, Wageningen, The Netherlands; 2 Department of Veterinary Biosciences, Faculty of Veterinary Medicine, University of Helsinki, Helsinki, Finland; 3 DOE Joint Genome Institute, Walnut Creek, California, United States of America; 4 Lawrence Livermore National Laboratory, Livermore, California, United States of America; 5 Los Alamos National Laboratory, Los Alamos, New Mexico, United States of America; The University of Maryland, United States of America

## Abstract

**Background:**

The human gastrointestinal tract contains a complex community of microbes, fulfilling important health-promoting functions. However, this vast complexity of species hampers the assignment of responsible organisms to these functions. Recently, *Akkermansia muciniphila*, a new species from the deeply branched phylum *Verrucomicrobia*, was isolated from the human intestinal tract based on its capacity to efficiently use mucus as a carbon and nitrogen source. This anaerobic resident is associated with the protective mucus lining of the intestines.

**Methodology/Principal Findings:**

In order to uncover the functional potential of *A. muciniphila*, its genome was sequenced and annotated. It was found to contain numerous candidate mucinase-encoding genes, but lacking genes encoding canonical mucus-binding domains. Numerous phage-associated sequences found throughout the genome indicate that viruses have played an important part in the evolution of this species. Furthermore, we mined 37 GI tract metagenomes for the presence, and genetic diversity of *Akkermansia* sequences. Out of 37, eleven contained 16S ribosomal RNA gene sequences that are >95% identical to that of *A. muciniphila*. In addition, these libraries were found to contain large amounts of *Akkermansia* DNA based on average nucleotide identity scores, which indicated in one subject co-colonization by different *Akkermansia* phylotypes. An additional 12 libraries also contained *Akkermansia* sequences, making a total of ∼16 Mbp of new *Akkermansia* pangenomic DNA. The relative abundance of *Akkermansia* DNA varied between <0.01% to nearly 4% of the assembled metagenomic reads. Finally, by testing a large collection of full length 16S sequences, we find at least eight different representative species in the genus *Akkermansia*.

**Conclusions/Significance:**

These large repositories allow us to further mine for genetic heterogeneity and species diversity in the genus *Akkermansia*, providing novel insight towards the functionality of this abundant inhabitant of the human intestinal tract.

## Introduction

Humans host a vast variety of microorganisms associated with the various body surfaces, such as on their skin [1] as well as in their gastrointestinal (GI) tract [2], [3]. However, only a minor fraction has been shown amenable to cultivation [4]. One way of probing the diversity of commensals and mutualists in the GI tract microbiota is through metagenomics. This culture-independent approach can capture for example the default proxies for species richness, the 16S ribosomal RNA (rRNA) sequences [5]. Subsequent analyses allow the quantification of the differences in colonization diversity between individuals, as well as their overlapping core microbiota [6], [7]. Alternatively, attempts have been made to sequence all microbial DNA of different individuals [8], now providing extensive gene catalogues of the human GI tract microbiome [9].

Due to its high phylogenetic and functional diversity, the GI tract microbial ecosystem represents a virtual organ that performs an array of health-promoting functions, from metabolizing otherwise inaccessible foods, the storage of fat, to the production of important vitamins [10], [11], [12], [13]. However, determining which species is responsible for what function can be an arduous task, since relatively little of the microbial diversity has been functionally characterized. This frustrates our understanding of the relationships with and between the residents of the microbiota.

Recently, *Akkermansia muciniphila*, a novel representative of the deeply rooted phylum *Verrucomicrobia*, was isolated from the human GI tract [14]. *A. muciniphila* was isolated using mucin, a complex glycosylated protein, which is used as a sole carbon and nitrogen source. Mucin is the major component of the protective coating of the human intestinal epithelium, where bacteria live in close proximity to human cells [15], [16]. The Gram-negative anaerobe *A. muciniphila* is known to colonize a substantial part of the human population, starting at early childhood [17], and by adulthood reaching densities up to 3% (∼1×10^9^) of the total bacterial count in feces [18]. Recently, *A. muciniphila* has been found to be inversely related to the severity of appendicitis [19], as well as being present in lower numbers in patients with inflammatory bowel disease [20], providing first glimpses of its association with human health issues.

Except for the significant colonization of *A. muciniphila*, little is known about this frequent and abundant resident of the GI tract. Thus far, only a few genomes of *Verrucomicrobiae* are available, hampering insight into the evolutionary history of this phylum. In order to uncover the functional capacity of *A. muciniphila*, we sequenced the complete genome of this species. Its full genetic repertoire is key in understanding the role of this abundant colonizer. Furthermore, by probing available GI tract metagenomic libraries with the full genome we shed light on the abundance and diversity of *Akkermansia*.

## Results

### General characteristics of the genome

The complete genome of *A. muciniphila* ATCC BAA-835 is composed of one circular chromosome of 2,664,102 bp with an average G+C content of 55.8%. The genome has a total of 2,176 predicted protein-coding sequences, with an overall coding capacity of 88.8%. Of the predicted protein-coding genes, 1,408 (65%) could be assigned a putative function, whereas 768 (35%) encode hypothetical proteins, with 38 (1.7%) of all protein-coding genes classified as pseudogenes.

Comparision to the six other available full and draft genome sequences of representatives of the *Verrucomicrobia* phylum showed that *A. muciniphila* shares 28.8%, 24.5%, 19.8% 17.9%, 16.0%, and 14.6%, coding sequences (CDS) with *Verrucomicrobium spinosum* DSM 4136, *Chthoniobacter flavus Ellin428*, *Pedosphaera parvula Ellin514*, *Opitutus terrae* PB90-1, *Methylacidiphilum infernorum V4 and Opitutaceae bacterium TAV2*, respectively. Overall, the available verrucomicrobial genomes show large variations in their GC content and genome size. A brief summary of the main characteristics of these seven genomes is provided in Table 1.

**Table 1:** Characteristics of verrucomicrobial genome sequences.

Genome	Total size (bp)	GC %	Coding Capacity (%)	Genes assigned to COG	Predicted ORFs	Genes encoding signal peptides	Number of CRISPR loci	Number of phage-associated sequences
*Akkermansia muciniphila* ATCC BAA-835	2664102	55.8	88.8	1489	2176	567 (26.1%)	2	9
*Verrucomicrobium spinosum* DSM 4136	8220857	60.3	86.0	3433	6509	1788 (27.5%)	3	14
*Methylacidiphilum infernorum* V4	2287145	45.5	91.2	1449	2472	330 (13.4%)	4	0
*Opitutus terrae* PB90-1	5957605	65.3	89.0	3102	4632	1428 (30.8%)	0	6
*Pedosphaera parvula* Ellin514	7474933	52.6	89.0	3508	6402	1857 (29.0%)	0	4
*Opitutaceae bacterium* TAV2	4954527	60.5	84.3	2313	4036	1032 (25.6%)	1	21
*Chthoniobacter flavus* Ellin428	7848700	61.1	88.4	3658	6716	2007 (29.9%)	0	7

Further analysis of the COG distribution of verrucomicrobial genomes shows overall a similar trend in the relative abundance of genes in the main COG categories for the genomes of *A. muciniphila* and *Methylacidiphilum infernorum* V4, including a more than average occurrence of genes in classes “Coenzyme metabolism” (H), “Nucleotide transport and metabolism” (F) and “Translation, ribosomal structure and biogenesis” (J), whereas relative abundance of genes is lower in categories “Transcription”(K) and “Signal transduction mechanisms”(T) in comparison to other verrucomicrobial genomes (Table S1). Furthermore, a subsequent comparison of the COG distribution of all *A. muciniphila* genes as well as 1337 *A. muciniphila* specific genes absent in the other six verrucomicrobial genomes revealed that those related to “Carbohydrate transport and metabolism” (G) and “Cell envelope biogenesis, outer membrane” (M) categories were enriched in the fraction of *A. muciniphila* specific genes. In contrast categories “Translation, ribosomal structure and biogenesis” (J) and “Nucleotide transport and metabolism” (F) were underrepresented in *A. muciniphila* specific genes.

In line with the above, further inspection of the annotated genome showed that *A. muciniphila* is predicted to synthesize all 20 canonical amino acids, as well as important co-factors and vitamins (data not shown), indicating that development of defined synthetic media for future post-genomic studies should be feasible. Furthermore, genome analysis suggested the ability to metabolize a variety of carbohydrates previously not found to be metabolized, such as galactose, cellobiose, melobiose and fructose [14], and will be addressed by ongoing efforts towards the generation and experimental validation of a genome-based metabolic models.

A large proportion (26%, 567 proteins) of the predicted *A. muciniphila* proteome contains a signal peptide cleavage site as predicted by signalP [21]. This seems to be a general trend for the *Verrucomicrobia* (Table S2). From this putative secretome, 61 proteins (11%) are annotated as glycosyl hydrolases, proteases, sulfatases and sialidases (35, 13, 11 and 2, respectively), and therefore strong candidates to be involved in the degradation of mucin. A substantial fraction of all proteins that are predicted to be secreted, are hypothetical proteins (242; 43%), a number of which may also be involved in mucin degradation and processing. Remarkably, no canonical mucus-binding domains are encountered in the proteome of *A. muciniphila*, and therefore no candidates involved in the adherence to the mucus layer of the host via these domains [22]. However, a recent study identified a novel module termed BACON (Bacteroidetes-associated carbohydrate-binding Often N-terminal) [23], which is also found in two *A. muciniphila* candidate mucinases (encoded by Amuc_0953, a sulfatase, and Amuc_2164, a glycosyl hydrolase), and is thought to be involved in mucin binding. But in contrast to most BACON-motif containing proteins, the two *Akkermansia* proteins have the motif on the C-terminus. Finally, a novel C-terminal targeting signal (TIGR02595) was recently identified in proteins from a variety of mainly Gram-negative species, the PEP-CTERM sequence consisting of a near invariant C-terminal Pro-Glu-Pro motif [24]. As was predicted, this motif could be found in 21 *A. muciniphila* proteins, encoded by genes scattered across the genome, and the corresponding exosortase EpsH (encoded by Amuc_1470), together forming a protein sorting system associated with exopolysaccharide expression.

Clustered regularly interspaced palindromic repeats (CRISPR) loci represent heritable and adaptive primitive immune systems in bacteria and archaea against invading agents such as bacteriophages or plasmids [25]. Two CRISPR loci are detected in the *A. muciniphila* genome, one in close proximity to a predicted mobile element (an integrase, encoded by Amuc_2006). These CRISPR loci 1 and 2 comprise direct repeats of 36 and 33 bp, and are interspersed 11 and 3 times with spacers, at coordinates 2438206–2438965 bp and 2507588–2507825 bp, respectively. Whereas the 36 bp repeat could not be classified, the 33 bp repeat is similar to repeat cluster 3 [26]. Homologues of CRISPR associated sequences *cas*1, *cas*2 and *csn*1 could be identified in close proximity to CRISPR locus 1 (Amuc_2008, Amuc_2009 and Amuc_2010). The predicted CRISPR locus 2, however, lacks proximal homologues to known CRISPR associated sequences. Due to the differences in repeat sequence and size it is unclear whether both repeat loci can be processed by the *cas* system located near CRISPR locus 1. In addition to these CRISPR loci, the presence of 9 predicted phage-related sequences (Amuc_0323, Amuc_0551, Amuc_1116, Amuc_1335, Amuc_1348, Amuc_1355, Amuc_1367, Amuc_1711 and Amuc_2017) suggests that *A. muciniphila* experienced frequent infection by bacteriophages.

Recently, the human microbiota were found to be a natural reservoir for antibiotic resistance genes [27]. As a frequent and abundant human resident, we queried the *A. muciniphila* genome for possible antibiotic resistance associated genes. We found potential beta-lactamase genes in the genome (Amuc_0106 and Amuc_0183), belonging to beta-lactamases classes C and A, respectively, as well as a gene coding for a 5-nitroimidazole antibiotic resistance protein (Amuc_1953). Furthermore, the *A. muciniphila* genome contains a gene that codes for a putative secreted antibiotic biosynthesis monooxygenase (Amuc_1805, PFAM PF03992).

Long mononucleotide repeats in *A. muciniphila* are overrepresented at the gene termini (Table S3 and Figure S1), as found previously for archaea, bacteria and eukaryotes [28], [29]. These repeats are known to be involved in prokaryotic transcriptional or translational phase variation [30]. Long homopolymeric tracts of >8 bp are found in 17 genes in *A. muciniphila*, amongst which 2 genes involved in the capsular polysaccharide biosynthesis; a capsular exopolysaccharide biosynthesis gene with a (G)_8_ repeat, and a gene that codes for an acyltransferase with a (C)_10_ repeat (Amuc_1413 and Amuc_2098, respectively).

Images of *A. muciniphila* have shown that the cells are frequently covered with flagella-like structures [14]. However, no obvious candidate genes have been discerned in the genome that could encode the putative proteinaceous building blocks of these filaments. Recently, studies into *Lactobacillus rhamnosus* have shown that this bacterium contains pili that are indispensible for interactions with human mucus [31]. In *A. muciniphila*, these structures are therefore interesting targets for proteomic investigation, since the availability of the genome sequence enables straightforward determination of the amino acid composition of extracellular proteins.

### Mining for *Akkermansia* DNA in metagenomic libraries of human GI tracts

Previous studies have shown that *A. muciniphila* is a common and abundant colonizer of the human GI tract, detectable in approximately 75% of the human population [17]. Therefore, we have queried 37 metagenomic libraries from an international effort in the cataloguing of human GI tract microbiomes (M. Arumugam et al. *under revision*) for the presence of *A. muciniphila* 16S rRNA gene sequences. Eleven (30%) of the 37 libraries contained sequences >95% identical to the *A. muciniphila* 16S rRNA gene query (Table 2, Table S4). In most cases, the nucleotide identity was >99% (ambiguous nucleotides excluded), except in one case (Italian male, 87 years old, subject B), where a complete 16S rRNA gene locus was identified with only 98% identity to that of *A. muciniphila*.

**Table 2:** General characteristics of the 37 metagenomes used in this study (metagenomes that contain predicted *Akkermansia* sequences are indicated in bold).

#	Sample	Counts	Total size	Amount of Akkermansia DNA (>200 bp, >90% identity)	Number of contigs	Relative abundance (%)	HitChip relative abundance (%)**	Number of 16S rRNA hits (% identity)*
**1**	**A**	**57133**	**49201030**	**211602**	**275**	**0.43**		
**2**	**B**	**59564**	**53016574**	**155799**	**107**	**0.29**		**1 (98.14)**
**3**	**C**	**73824**	**61489938**	**310164**	**428**	**0.5**		
4	CD1	65088	63576076	0			0.017	
**5**	**CD2**	**85009**	**70601731**	**17168**	**25**	**0.02**	**0.015**	
6	D	38297	36284293	0				
**7**	**E**	**77082**	**63135227**	**1384051**	**1410**	**2.19**		**1 (99.58%)**
**8**	**F1-S**	**31096**	**35575967**	**22174**	**24**	**0.06**		
9	F1-T	37749	39020190	0				
10	F1-U	17588	22815676	0				
**11**	**F2-V**	**37793**	**41313262**	**35594**	**39**	**0.09**		
**12**	**F2-W**	**31171**	**36156855**	**6826**	**9**	**0.02**		
**13**	**F2-X**	**31685**	**34467680**	**309012**	**327**	**0.9**		
**14**	**F2-Y**	**36803**	**41718743**	**54428**	**58**	**0.13**		
**15**	**G**	**75435**	**62138255**	**195493**	**267**	**0.31**		**1 (99.59%)**
16	In-A	21092	24491884	0				
17	In-B	6791	10687920	0				
18	In-D	38642	39888261	0				
19	In-E	15971	19473697	0				
**20**	**In-M**	**17802**	**23882918**	**575**	**1**	**0**		
21	In-R	34389	38225044	0				
**22**	**MH12**	**110201**	**93505669**	**1881572**	**1560**	**2.01**	**0.055**	**1 (99.93%)**
**23**	**MH13**	**99166**	**83354756**	**2538185**	**1102**	**3.05**	**0.127**	**1 (99.87%)**
24	MH30	113540	96152661	0			0.032	
**25**	**MH6**	**105516**	**86753488**	**10407**	**16**	**0.01**	**0.017**	
**26**	**NO1**	**81876**	**67366915**	**524000**	**678**	**0.78**		**1 (100%)**
**27**	**NO3**	**73590**	**57125037**	**2253202**	**1398**	**3.94**		**1 (99.87%)**
**28**	**NO4**	**70331**	**57189273**	**1577353**	**1565**	**2.76**		**2 (100%, 99.81%)**
**29**	**NO8**	**87546**	**67187941**	**620695**	**793**	**0.92**		
30	OB1	78155	59651934	0				
31	OB2	83931	66168175	0				
**32**	**OB6**	**71160**	**57081404**	**585486**	**796**	**1.03**		**4 (100%, 100%, 100%, 98.33%)**
**33**	**OB8**	**77225**	**57168299**	**942798**	**1201**	**1.65**		**2 (99.84%, 98.75%)**
34	Subject7	41831	46136049	0				
35	Subject8	37448	46003405	0				
**36**	**UC4**	**98656**	**79886227**	**747**	**1**	**0**	**0.017**	
**37**	**UC6**	**115558**	**94544463**	**2294676**	**1409**	**2.43**	**0.017**	**1 (99.80%)**
			**Total**	**15,932,007**	**13,489**			

For the full table, see Table S6.

*) See also Table S4.

**) For the HITChip analyses, only the eight samples part of the MetaHIT project were tested.

Subsequently, we queried each of the metagenomic libraries with the entire genomic complement in order to discern all *Akkermansia* carriers in this dataset. The combined set of bacterial and archaeal genome sequences at NCBI (1026 genomes, obtained 22-01-2010) failed to show any non-*Akkermansia* hit with a nucleotide identity score >90% for over 200 bp when queried with the *A. muciniphila* genome (rRNA sequences excluded, data not shown). Therefore, applying these values as a conservative cut-off, we identified putative *Akkermansia* DNA in a further 12 metagenomic databases (Table 2, all predicted *Akkermansia* contigs are listed in Table S5), which brings the total of *Akkermansia* containing libraries up to 23 (62%). The 11 libraries that contained *Akkermansia* 16S rRNA gene sequences were found to contain on average over 1.3 Mbp of *Akkermansia* DNA, compared to an average of 133 kbp of *Akkermansia* DNA in the 12 libraries lacking the ribosomal proxy. The amount of *Akkermansia* DNA per database varied from a single contig of 575 bp (Japanese female, 4 months old, subject In-M), to well over 2.5 Mbp in 1102 contigs (Danish healthy male, 54 years old, subject MH13). The largest relative amount of *Akkermansia* DNA, 3.9% of the total assembled DNA, was found in a 61-year-old French healthy male (Subject NO3). In total 13,589 contigs, comprising 15.9 Mbp of novel *Akkermansia* sequences, were identified in over 1.98 Gbp of assembled metagenomic data.

**Figure 1:**
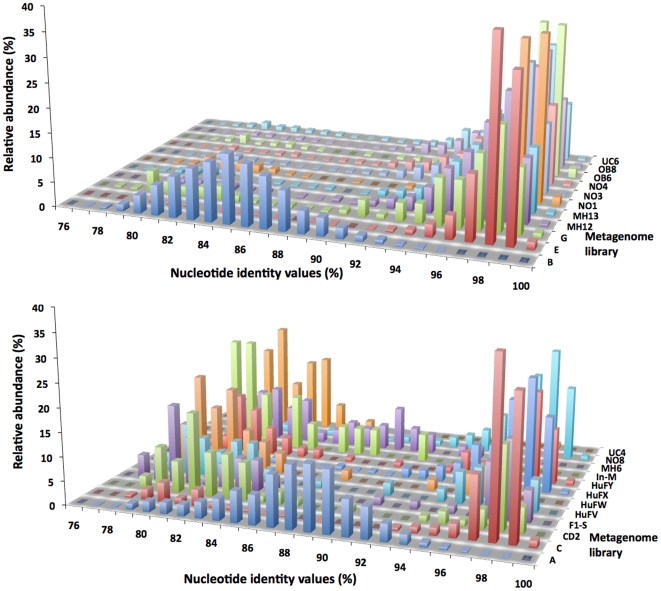
Average nucleotide identity (ANI) distributions for the 11 metagenomic libraries in which we found *Akkermansia*-like 16S rRNA sequences (1A) and for the 12 metagenomic libraries in which we did not find *Akkermansia*-like 16S rRNA sequences (1B), but which did show numerous hits with the *A. muciniphila* genome with nucleotide identity scores above 90%. Please note the distinctive distribution of ANI values of libraries B, A and MH6. Relative abundance scores (z-axis) are calculated for the total number of contigs per metagenome that show nucleotide identity scores >75%.

The average nucleotide identity (ANI) has been proposed to advance the definition of species boundaries in prokaryotes [32]. For each metagenomic database that contains over ten predicted *Akkermansia* sequences (i.e., stretches of >200 bp with >90% ANI), we analyzed the distribution of ANI scores for all contigs that we predict to be derived from *Akkermansia* (Figure 1). This shows that for most of the metagenomic libraries (20 out of 23 metagenomes), these contigs have an ANI of around 98% as compared to the *A. muciniphila* genome, and the subjects could be considered to be *A. muciniphila* carriers. However, metagenomic datasets from three individuals (A, B and MH6) display a distinctly different distribution of nucleotide identity values, with a much lower ANI (84–88%, though MH6 has several peaks, Figure 1). This indicates that these subjects are likely to be colonized by uncultured and unknown representatives of the *Akkermansia* genus. Moreover, in databases A and B, (parts of) both BACON domain containing protein-coding genes are encountered, suggestive of a mucolytic potential in these different *Akkermansia* species.

All 14 databases that are devoid of *Akkermansia-*like 16S rRNA gene sequences and of sequences >200 bp that were >90% identical to *Akkermansia* DNA, contained contigs with nucleotide identity scores between 75 and 90% and an average ANI of 80%. These sequences could belong to other species from the genus *Akkermansia*, though this is tentative.

In order to corroborate the possibility for co-colonization of individual microbiomes by different species of *Akkermansia*, we queried 9773 nearly full-length 16S rRNA sequences from a microbiome study in lean and obese twins [6], showing that out of 30 sampled individuals, 15 contain sequences with over 95% identity with the *A. muciniphila* 16S rRNA sequence (Table S7). One individual (TS148) harbours only 16S rRNA sequences with <98% identity, indicative of colonization by an unknown species from the *Akkermansia* genus, whereas four individuals (TS1, TS6, TS51 and TS150) harbour both *A. muciniphila* and other *Akkermansia* spp. 16S rRNA sequences, suggesting simultaneous colonization of these hosts by different species from this genus. Using these sequences we find a total of eight different species (each represented by at least two individual sequence traces) in the genus *Akkermansia* using an identity threshold of 98%, suggesting a large still unexplored intrageneric diversity, of which representatives also colonize human microbiomes.

## Discussion

We sequenced the genome of the human gut colonizer *Akkermansia muciniphila*, a representative of the phylum *Verrucomicrobia*. *A. muciniphila* has been isolated in basal medium using mucin as a sole carbon and nitrogen source [14], showing that this species is able to degrade the major component of the mucosal lining of the GI tract. Analyses of the distribution of this species have shown it to be a frequent and abundant colonizer of GI tracts in a range of animal hosts [33]. A further exploration into the functional roles of *A. muciniphila* in the GI tract, however, would be greatly facilitated by the availability of its genetic repertoire.

In genomic terms, *A. muciniphila* is an average member of the *Verrucomicrobia*, both in respect to the number of protein coding genes and the GC percentage of the genome, which fall between the values of the sequenced genomes of other members of this phylum. Verrucomicrobial genomes seem to contain a relatively large number of genes encoding a signal peptide when compared to other phyla, and correspondingly, in *A. muciniphila* approximately a quarter of the proteins encoded in the genome contain a signal peptide, and are therefore potentially secreted. Several of these proteins are predicted to be involved in the different steps of mucin degradation, whereas the large number of genes encoding signal-peptide bearing hypothetical proteins suggests that there may be a large undiscovered capacity of *A. muciniphila* to break down extracellular polymeric substrates, including mucin. Future studies, including proteomic analyses and functional screening of genomic libraries, can be expected to shed light on the involved enzymes, since recent analysis have confirmed the mucolytic activity of *Akkermansia* [34].

As found for other, mainly human-associated, organisms such as *Neisseria* spp., *Haemophilus* spp., *Campylobacter* spp. and *Helicobacter* spp., phase variation via mononucleotide repeat slippage may be employed by *A. muciniphila*. Notably, two genes involved in capsule synthesis contain very long repeats of guanines, which are known to be more severely underrepresented in the coding parts of genome sequences [35]. Capsules are known antigens encoded by numerous human pathogens [36], but another role of capsules is protection against desiccation [37]. This may be involved in the transmission of *Akkermansia* via the fecal-oral route.

The presence of two dinstinct CRISPR loci [38], as well as numerous presumably phage-derived sequences in the genome, suggests that viral infections have played an important part in the evolutionary history, and perhaps speciation, of *A. muciniphila*. Little was known about the variety of the *Akkermansia* strains and species that colonize a single microbiome, but the current analyses suggest that at least eight different species of the *Akkermansia* genus colonize the GI tracts of humans, and even simultaneous colonization by different species seems to take place. Whether this means that distinct niches exist for different specialist mucin-degraders in the GI tract, or whether humans are infected continuously by different *Akkermansia* species, resulting in discontinuous (co-)colonization, is unknown.

In three libraries, we encountered divergent *Akkermansia* sequences, based on low ANI values compared to the sequenced *A. muciniphila* genome. It is not possible with the current datasets to confirm whether these three species are identical to each other, since for two of these databases the *Akkermansia* 16S rRNA gene sequences are lacking in the metagenome. It is, however, tempting to speculate that these other *Akkermansia* species can also thrive on mucin as a carbon source, based on the presence of the BACON domain containing protein-coding genes, and therefore occupy a similar, if not the same, niche as *A. muciniphila*.

We approached the investigation of metagenomic libraries with a given complete genome as a query sequence. This increases, as expected, the detection of closely related strains and species in large metagenomes, and aids in the quantification of bacterial abundance. Many metagenomic repositories contain assembled DNA sequencing reads, which may skew the interpretation of the actual abundance of the organism of interest as opposed to the total number of raw sequence reads. However, hybridization signal strength in phylogenetic microarray analyses identified the same metagenome library with the largest amount of *Akkermansia* DNA. Further investigations into the congruence between different abundance estimates may help to validate their applicability.

Together, we present the genome sequence of *Akkermansia muciniphila*, as well as a number of its features that may be important in its ecology and evolution tuned to its niche, the human GI tract. These data enable a further characterization into the functional role of this abundant human-associated commensal.

## Materials and Methods

### DNA isolation

A glycerol stock of the *Akkermansia muciniphila* type strain (ATCC BAA-835) was inoculated in 500 ml anoxic basal medium containing pork gastric mucin as carbon and energy source and subsequently incubated at 37°C overnight as described previously [14]. Cells were harvested by centrifugation and used for high molecular weight DNA isolation using the standard Bacterial genomic DNA isolation using CTAB method recommended by the DOE Joint Genome Institute (JGI, Walnut Creek, CA) with minor modifications. In short, cells were resuspended in 14.8 ml modified TE (10 mM tris; 20 mM EDTA, pH 8.0). The modified TE has shown to prevent DNA degradation (data not shown). Subsequently, cells were lyzed using lysozyme and proteinase K, and DNA was extracted and purified using CTAB and phenol∶chloroform∶isoamylalcohol extractions. After precipitation in 2-propanol and washing in 70% ethanol, the DNA was resuspended in 400 µl TE containing 40 µg RNase A. Following quality and quantity check using agarose gel electrophoresis in the presence of ethidium bromide, and spectrophotometric measurement using a NanoDrop ND-1000 spectrophotometer (NanoDrop® Technologies, Wilmington, DE, USA), respectively, the DNA was precipitated in 2-propanol and shipped to the JGI for whole genome shotgun sequencing.

### Genome sequencing and assembly

The genome of *Akkermansia muciniphila* was sequenced at the JGI using a combination of 3 kb, 8 kb and 40 kb (fosmid) DNA libraries. In addition to Sanger sequencing, 454 pyrosequencing was performed to a depth of 20× coverage. All general aspects of library construction and sequencing performed at the JGI can be found at http://www.jgi.doe.gov/. Draft assemblies were based on 51,010 total reads and resulted in approximately 15.5× coverage of the genome. The Phred/Phrap/Consed software package (www.phrap.com) was used for sequence assembly and quality assessment [39], [40], [41]. Gaps between contigs were closed by custom primer walks on gap spanning clones or PCR products. A total of 567 additional reactions were necessary to close gaps and to raise the quality of the finished sequence. The completed genome sequence of *A. muciniphila* contains 50,774 reads, achieving an average of 17.7-fold sequence coverage per base with an error rate less than 1 in 100,000.

### Gene calling

The gene modeling program Prodigal (http://prodigal.ornl.gov/) was run on the finished genome, using default settings that permit overlapping genes and using ATG, GTG, and TTG as potential starts. The resulting protein translations were compared to Genbank's non-redundant database (NR), the Swiss-Prot/TrEMBL, PRIAM, Pfam, TIGRFam, Interpro, KEGG, and COGs databases using BLASTP or HMMER. From these results, product assignments were made. Initial criteria for automated functional assignment set priority based on PRIAM, TIGRFam, Pfam, Intepro profiles, pairwise BLAST vs Swiss-Prot/TrEMBL, KEGG, and COG groups. Manual corrections to automated functional assignments were completed on an individual gene-by-gene basis as needed. The annotation was imported into The Joint Genome Institute Integrated Microbial Genomes (IMG; http://img.jgi.doe.gov/cgi-bin/pub/main.cgi) [42]. Singleton identification was carried out as described by Blom et al. [43]. Genes from other available verrucomicrobial genomes were assigned to COGs using RPS-BLAST (Reverse Position Specific BLAST) and NCBI's Conserved Domain Database (CDD). Top hits were taken with an E-value cut-off of 10^−2^. The *A. muciniphila* genome sequence is available at NCBI under accession number NC_010655.

### Metagenome mining

The GI tract metagenomes originate from previous studies, all based on Sanger sequencing, and have been re-processed with the SMASH pipeline [44]. General characteristics of these metagenomes are given in Table 2 and Table S6. These 37 metagenomes were queried with the *A. muciniphila* 16S rRNA gene sequence or with its entire genome sequence using BLAST [45], with hits required to be over 200 bp in length and with over 90% nucleotide identity (rRNA regions were filtered out in the whole genome BLAST analyses).

Nearly full-length 16S rRNA sequences from a twin microbiome study [6] were included (FJ362604–FJ372382; 9773 were extracted from NCBI) for co-colonization analyses and species determination. Different species were assigned using a 98% sequence identity cut-off threshold, and each species group requires at least two representatives.

## Supporting Information

Figure S1 **Positional bias of homopolymeric repeats within all protein coding genes from** ***Akkermansia muciniphila*** **.** All genes were divided proportionally into five quintiles (with at its 5′ end Quintile 1, next Quintile 2, Quintile 3 and Quintile 4, and Quintile 5 as the 3′ end). With increasing repeat length (from >4 than >7 nucleotides), the repeats are progressively more abundant in the first quintile. Percentages are depicted as deviations relative to the expected value of 20% per gene quintile for a non-biased intragenic distribution of repeats.
(TIFF)

Table S1 **COG assignments for the seven verrucomicrobial genomes (for full names, see** **Table 2** **).**
(DOCX)

Table S2 **SignalP predictions for a range of bacterial phyla and species (based on JGI predictions and curations).** In bold, the phylum-averages are depicted.
(DOCX)

Table S3 **List of** ***Akkermansia muciniphila*** **protein coding genes that include mononucleotide repeats of 9 bp or longer.** The relative gene position (between 0 and 1) is calculated based on the start (relative gene position 0) and end (relative gene position of 1) of each gene.
(DOCX)

Table S4 **The presence of 16S ribosomal sequences (>95% identical to that of** ***Akkermansia muciniphila*** **) in the metagenomic databases.**
(DOCX)

Table S5 **Lists of predicted** ***Akkermansia*** **contigs for the 37 metagenomic databases.**
(XLSX)

Table S6 **General characteristics of the 37 metagenomes used in this study (metagenomes that contain predicted** ***Akkermansia*** **sequences are indicated in green).**
(DOCX)

Table S7 **Overview of** ***Akkermansia*** **-like sequences (>95% identity and <98% identity compared to the sequenced** ***A. muciniphila*** **16S sequence) in 9773 nearly full-length 16S rRNA sequences from a microbiome study in twins** [**6**] **.**
(DOCX)

## References

[1] Grice EA, Kong HH, Conlan S, Deming CB, Davis J (2009). Topographical and temporal diversity of the human skin microbiome.. Science.

[2] Pei Z, Bini EJ, Yang L, Zhou M, Francois F (2004). Bacterial biota in the human distal esophagus.. Proc Natl Acad Sci U S A.

[3] Eckburg PB, Bik EM, Bernstein CN, Purdom E, Dethlefsen L (2005). Diversity of the human intestinal microbial flora.. Science.

[4] Zoetendal EG, Rajilic-Stojanovic M, de Vos WM (2008). High-throughput diversity and functionality analysis of the gastrointestinal tract microbiota.. Gut.

[5] Gill SR, Pop M, Deboy RT, Eckburg PB, Turnbaugh PJ (2006). Metagenomic analysis of the human distal gut microbiome.. Science.

[6] Turnbaugh PJ, Hamady M, Yatsunenko T, Cantarel BL, Duncan A (2009). A core gut microbiome in obese and lean twins.. Nature.

[7] Tap J, Mondot S, Levenez F, Pelletier E, Caron C (2009). Towards the human intestinal microbiota phylogenetic core.. Environ Microbiol.

[8] Kurokawa K, Itoh T, Kuwahara T, Oshima K, Toh H (2007). Comparative metagenomics revealed commonly enriched gene sets in human gut microbiomes.. DNA Res.

[9] Qin J, Li R, Raes J, Arumugam M, Burgdorf KS (2010). A human gut microbial gene catalogue established by metagenomic sequencing.. Nature.

[10] Backhed F, Ding H, Wang T, Hooper LV, Koh GY (2004). The gut microbiota as an environmental factor that regulates fat storage.. Proc Natl Acad Sci U S A.

[11] Backhed F, Ley RE, Sonnenburg JL, Peterson DA, Gordon JI (2005). Host-bacterial mutualism in the human intestine.. Science.

[12] Hehemann JH, Correc G, Barbeyron T, Helbert W, Czjzek M (2010). Transfer of carbohydrate-active enzymes from marine bacteria to Japanese gut microbiota.. Nature.

[13] Klaassens ES, Boesten RJ, Haarman M, Knol J, Schuren FH (2009). Mixed-species genomic microarray analysis of fecal samples reveals differential transcriptional responses of bifidobacteria in breast- and formula-fed infants.. Appl Environ Microbiol.

[14] Derrien M, Vaughan EE, Plugge CM, de Vos WM (2004). Akkermansia muciniphila gen. nov., sp. nov., a human intestinal mucin-degrading bacterium.. Int J Syst Evol Microbiol.

[15] Lievin-Le Moal V, Servin AL (2006). The front line of enteric host defense against unwelcome intrusion of harmful microorganisms: mucins, antimicrobial peptides, and microbiota.. Clin Microbiol Rev.

[16] Derrien M, van Passel MWJ, van de Bovenkamp JHB, Schipper R, de Vos WM (2010). Mucin-bacterial interactions in the human oral cavity and digestive tract.. Gut Microbes.

[17] Collado MC, Derrien M, Isolauri E, de Vos WM, Salminen S (2007). Intestinal integrity and Akkermansia muciniphila, a mucin-degrading member of the intestinal microbiota present in infants, adults, and the elderly.. Appl Environ Microbiol.

[18] Derrien M, Collado MC, Ben-Amor K, Salminen S, de Vos WM (2008). The Mucin degrader Akkermansia muciniphila is an abundant resident of the human intestinal tract.. Appl Environ Microbiol.

[19] Swidsinski A, Dorffel Y, Loening-Baucke V, Theissig F, Ruckert JC (2011). Acute appendicitis is characterised by local invasion with Fusobacterium nucleatum/necrophorum.. Gut.

[20] Png CW, Linden SK, Gilshenan KS, Zoetendal EG, McSweeney CS (2010). Mucolytic Bacteria With Increased Prevalence in IBD Mucosa Augment In Vitro Utilization of Mucin by Other Bacteria.. Am J Gastroenterol.

[21] Bendtsen JD, Nielsen H, von Heijne G, Brunak S (2004). Improved prediction of signal peptides: SignalP 3.0.. J Mol Biol.

[22] Boekhorst J, Helmer Q, Kleerebezem M, Siezen RJ (2006). Comparative analysis of proteins with a mucus-binding domain found exclusively in lactic acid bacteria.. Microbiology.

[23] Mello LV, Chen X, Rigden DJ (2010). Mining metagenomic data for novel domains: BACON, a new carbohydrate-binding module.. FEBS Lett.

[24] Haft DH, Paulsen IT, Ward N, Selengut JD (2006). Exopolysaccharide-associated protein sorting in environmental organisms: the PEP-CTERM/EpsH system. Application of a novel phylogenetic profiling heuristic.. BMC Biol.

[25] van der Oost J, Jore MM, Westra ER, Lundgren M, Brouns SJ (2009). CRISPR-based adaptive and heritable immunity in prokaryotes.. Trends Biochem Sci.

[26] Kunin V, Sorek R, Hugenholtz P (2007). Evolutionary conservation of sequence and secondary structures in CRISPR repeats.. Genome Biol.

[27] Sommer MO, Dantas G, Church GM (2009). Functional characterization of the antibiotic resistance reservoir in the human microflora.. Science.

[28] van Passel MW, Ochman H (2007). Selection on the genic location of disruptive elements.. Trends Genet.

[29] van Passel MW, de Graaff LH (2008). Mononucleotide repeats are asymmetrically distributed in fungal genes.. BMC Genomics.

[30] Saunders NJ, Jeffries AC, Peden JF, Hood DW, Tettelin H (2000). Repeat-associated phase variable genes in the complete genome sequence of Neisseria meningitidis strain MC58.. Mol Microbiol.

[31] Kankainen M, Paulin L, Tynkkynen S, von Ossowski I, Reunanen J (2009). Comparative genomic analysis of Lactobacillus rhamnosus GG reveals pili containing a human- mucus binding protein.. Proc Natl Acad Sci U S A.

[32] Konstantinidis KT, Tiedje JM (2005). Genomic insights that advance the species definition for prokaryotes.. Proc Natl Acad Sci U S A.

[33] Ley RE, Lozupone CA, Hamady M, Knight R, Gordon JI (2008). Worlds within worlds: evolution of the vertebrate gut microbiota.. Nat Rev Microbiol.

[34] Png CW, Linden SK, Gilshenan KS, Zoetendal EG, McSweeney CS (2010). Mucolytic Bacteria With Increased Prevalence in IBD Mucosa Augment In Vitro Utilization of Mucin by Other Bacteria.. Am J Gastroenterol.

[35] Ackermann M, Chao L (2006). DNA sequences shaped by selection for stability.. PLoS Genet.

[36] Roberts IS (1996). The biochemistry and genetics of capsular polysaccharide production in bacteria.. Annu Rev Microbiol.

[37] Ophir T, Gutnick DL (1994). A Role for Exopolysaccharides in the Protection of Microorganisms from Desiccation.. Appl Environ Microbiol.

[38] Barrangou R, Fremaux C, Deveau H, Richards M, Boyaval P (2007). CRISPR provides acquired resistance against viruses in prokaryotes.. Science.

[39] Ewing B, Hillier L, Wendl MC, Green P (1998). Base-calling of automated sequencer traces using phred. I. Accuracy assessment.. Genome Res.

[40] Ewing B, Green P (1998). Base-calling of automated sequencer traces using phred. II. Error probabilities.. Genome Res.

[41] Gordon D, Abajian C, Green P (1998). Consed: a graphical tool for sequence finishing.. Genome Res.

[42] Markowitz VM, Korzeniewski F, Palaniappan K, Szeto E, Werner G (2006). The integrated microbial genomes (IMG) system.. Nucleic Acids Res.

[43] Blom J, Albaum SP, Doppmeier D, Puhler A, Vorholter FJ (2009). EDGAR: a software framework for the comparative analysis of prokaryotic genomes.. BMC Bioinformatics.

[44] Arumugam M, Harrington ED, Foerstner KU, Raes J, Bork P (2010). SmashCommunity: a metagenomic annotation and analysis tool.. Bioinformatics.

[45] Altschul SF, Gish W, Miller W, Myers EW, Lipman DJ (1990). Basic local alignment search tool.. J Mol Biol.

