# Supplementary figures and images for: The Genome of Akkermansia muciniphila, a Dedicated Intestinal Mucin Degrader, and Its Use in Exploring Intestinal Metagenomes
Source: PLoS One. 2011 Mar 3;6(3):e16876. doi: 10.1371/journal.pone.0016876 (PMC3048395; doi:10.1371/journal.pone.0016876)

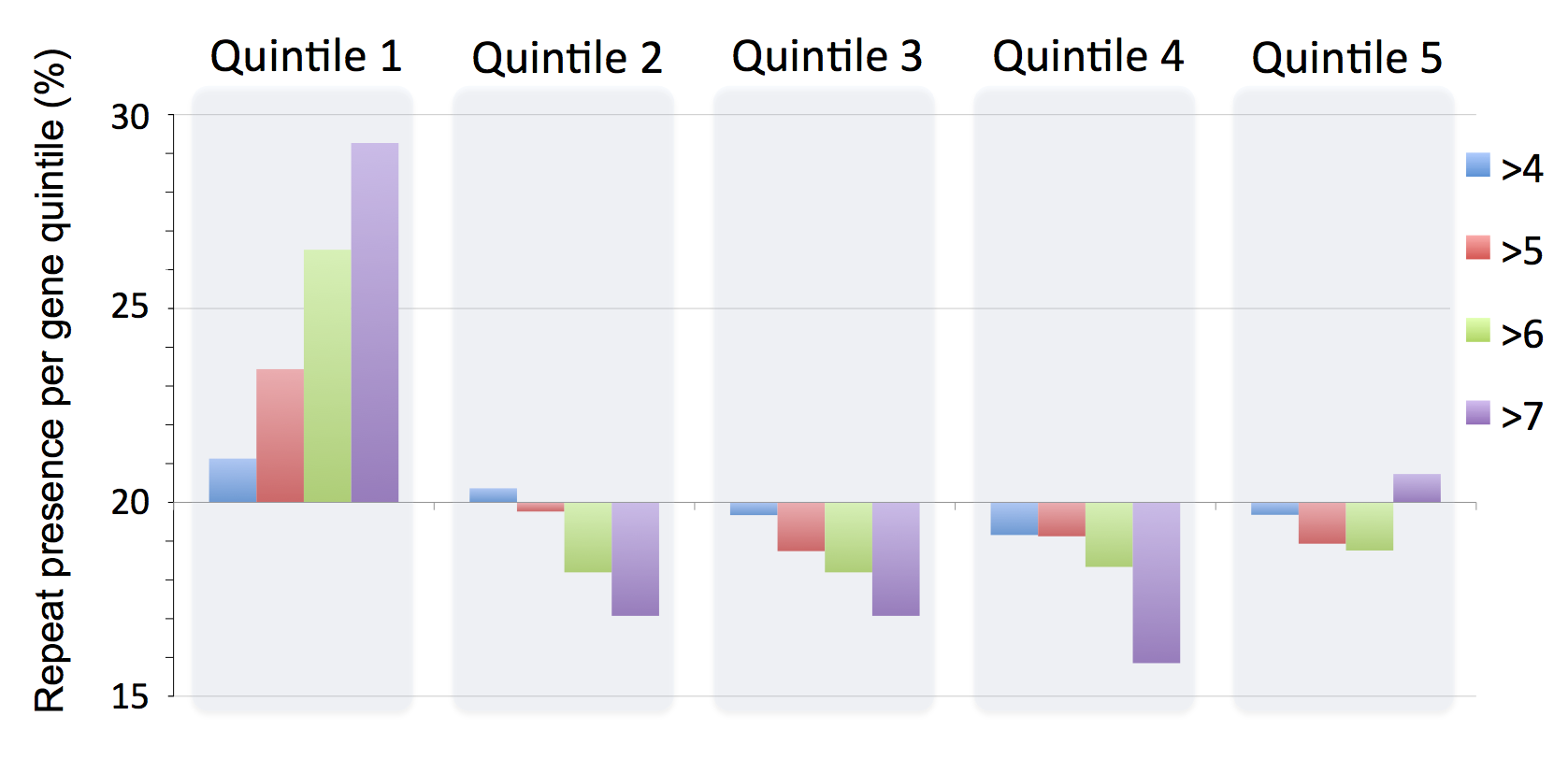

Supplement: Figure S1 — Positional bias of homopolymeric repeats within all protein coding genes from Akkermansia muciniphila . All genes were divided proportionally into five quintiles (with at its 5′ end Quintile 1, next Quintile 2, Quintile 3 and Quintile 4, and Quintile 5 as the 3′ end). With increasing repeat length (from >4 than >7 nucleotides), the repeats are progressively more abundant in the first quintile. Percentages are depicted as deviations relative to the expected value of 20% per gene quintile for a non-biased intragenic distribution of repeats. (TIFF) [file pone.0016876.s001.tiff]
