# Supplementary material for: The Genome of Akkermansia muciniphila, a Dedicated Intestinal Mucin Degrader, and Its Use in Exploring Intestinal Metagenomes
Source: PLoS One. 2011 Mar 3;6(3):e16876. doi: 10.1371/journal.pone.0016876 (PMC3048395; doi:10.1371/journal.pone.0016876)
Supplement: Table S1 — COG assignments for the seven verrucomicrobial genomes (for full names, see Table 2 ). (DOCX) [file pone.0016876.s002.docx]

Supplementary Table S1. COG assignments for the seven verrucomicrobial genomes (for full names, see Table 2).

| SYMBOL | COG Categories | Gene Count Ellin428 | % of Total 4217 | Gene Count Akkermansia | % of Total 1663 | Gene Count Opitutaceae bacterium TAV2 | % of Total 2707 | Gene Count Methylacidiphilum | % of Total 1591 | Gene Count Opitutus terrae PB90-1 | % of Total 3515 | Gene Count Ellin514 | % of Total 4119 | Gene Count Verrucomicrobium | % of Total 3914 |
| --- | --- | --- | --- | --- | --- | --- | --- | --- | --- | --- | --- | --- | --- | --- | --- |
| E | Amino acid transport and metabolism | 269 | 6.38 | 146 | 8.78 | 207 | 7.65 | 132 | 8.3 | 238 | 6.77 | 228 | 5.54 | 254 | 6.49 |
| G | Carbohydrate transport and metabolism | 276 | 6.54 | 112 | 6.73 | 213 | 7.87 | 96 | 6.03 | 300 | 8.53 | 276 | 6.7 | 238 | 6.08 |
| D | Cell cycle control, cell division, chromosome partitioning | 27 | 0.64 | 16 | 0.96 | 18 | 0.66 | 19 | 1.19 | 33 | 0.94 | 34 | 0.83 | 29 | 0.74 |
| N | Cell motility | 80 | 1.9 | 13 | 0.78 | 180 | 6.65 | 3 | 0.19 | 96 | 2.73 | 147 | 3.57 | 84 | 2.15 |
| M | Cell wall/membrane/envelope biogenesis | 325 | 7.71 | 156 | 9.38 | 142 | 5.25 | 146 | 9.18 | 254 | 7.23 | 305 | 7.4 | 322 | 8.23 |
| B | Chromatin structure and dynamics | 4 | 0.09 | 0 | 0 | 1 | 0.04 | 1 | 0.06 | 2 | 0.06 | 2 | 0.05 | 2 | 0.05 |
| H | Coenzyme transport and metabolism | 159 | 3.77 | 90 | 5.41 | 95 | 3.51 | 108 | 6.79 | 136 | 3.87 | 143 | 3.47 | 139 | 3.55 |
| Z | Cytoskeleton | 1 | 0.02 | 0 | 0 | 2 | 0.07 | 0 | 0 | 3 | 0.09 | 15 | 0.36 | 1 | 0.03 |
| V | Defense mechanisms | 91 | 2.16 | 40 | 2.41 | 47 | 1.74 | 25 | 1.57 | 113 | 3.21 | 95 | 2.31 | 83 | 2.12 |
| C | Energy production and conversion | 201 | 4.77 | 95 | 5.71 | 142 | 5.25 | 132 | 8.3 | 205 | 5.83 | 228 | 5.54 | 149 | 3.81 |
| S | Function unknown | 437 | 10.36 | 121 | 7.28 | 165 | 6.1 | 107 | 6.73 | 246 | 7 | 329 | 7.99 | 388 | 9.91 |
| R | General function prediction only | 535 | 12.69 | 198 | 11.91 | 284 | 10.49 | 166 | 10.43 | 384 | 10.92 | 531 | 12.89 | 518 | 13.23 |
| P | Inorganic ion transport and metabolism | 189 | 4.48 | 93 | 5.59 | 109 | 4.03 | 94 | 5.91 | 184 | 5.23 | 163 | 3.96 | 229 | 5.85 |
| U | Intracellular trafficking, secretion, and vesicular transport | 118 | 2.8 | 41 | 2.47 | 203 | 7.5 | 47 | 2.95 | 96 | 2.73 | 208 | 5.05 | 140 | 3.58 |
| I | Lipid transport and metabolism | 142 | 3.37 | 51 | 3.07 | 73 | 2.7 | 44 | 2.77 | 92 | 2.62 | 112 | 2.72 | 118 | 3.01 |
| F | Nucleotide transport and metabolism | 69 | 1.64 | 46 | 2.77 | 47 | 1.74 | 63 | 3.96 | 73 | 2.08 | 83 | 2.02 | 59 | 1.51 |
| O | Posttranslational modification, protein turnover, chaperones | 162 | 3.84 | 68 | 4.09 | 81 | 2.99 | 70 | 4.4 | 125 | 3.56 | 158 | 3.84 | 131 | 3.35 |
| A | RNA processing and modification | 1 | 0.02 | 0 | 0 | 0 | 0 | 0 | 0 | 0 | 0 | 0 | 0 | 2 | 0.05 |
| L | Replication, recombination and repair | 203 | 4.81 | 105 | 6.31 | 122 | 4.51 | 91 | 5.72 | 144 | 4.1 | 142 | 3.45 | 231 | 5.9 |
| Q | Secondary metabolites biosynthesis, transport and catabolism | 86 | 2.04 | 17 | 1.02 | 48 | 1.77 | 25 | 1.57 | 72 | 2.05 | 110 | 2.67 | 74 | 1.89 |
| T | Signal transduction mechanisms | 367 | 8.7 | 37 | 2.22 | 82 | 3.03 | 45 | 2.83 | 297 | 8.45 | 339 | 8.23 | 251 | 6.41 |
| K | Transcription | 316 | 7.49 | 78 | 4.69 | 319 | 11.78 | 53 | 3.33 | 259 | 7.37 | 309 | 7.5 | 316 | 8.07 |
| J | Translation, ribosomal structure and biogenesis | 159 | 3.77 | 140 | 8.42 | 127 | 4.69 | 124 | 7.79 | 163 | 4.64 | 162 | 3.93 | 156 | 3.99 |
|  | Not in COGs | 3120 | 46.03 | 749 | 33.47 | 1792 | 43.65 | 1072 | 42.52 | 1599 | 34.01 | 2963 | 45.79 | 3151 | 47.86 |
