# Supplementary material for: The Genome of Akkermansia muciniphila, a Dedicated Intestinal Mucin Degrader, and Its Use in Exploring Intestinal Metagenomes
Source: PLoS One. 2011 Mar 3;6(3):e16876. doi: 10.1371/journal.pone.0016876 (PMC3048395; doi:10.1371/journal.pone.0016876)
Supplement: Table S2 — SignalP predictions for a range of bacterial phyla and species (based on JGI predictions and curations). In bold, the phylum-averages are depicted. (DOCX) [file pone.0016876.s003.docx]

Supplementary Table S2. SignalP predictions for a range of bacterial phyla and species (based on JGI predictions and curations). In bold, the phylum-averages are depicted.

| **Phylum** | **Species** | **Genes coding signal peptides (%)** |
| --- | --- | --- |
| Actinobacteria | | **20.4** |
|  | *Corynebacterium urealyticum* DSM 7109. | 19.9 |
|  | *Frankia* sp. CcI3. | 18.7 |
|  | *Streptomyces griseus* subsp. *griseus* NBRC 13350. | 23.0 |
|  | *Mycobacterium bovis* AF2122/97. | 23.4 |
|  | *Arthrobacter aurescens* TC1. | 21.8 |
|  | *Propionibacterium acnes* KPA171202. | 21.5 |
|  | *Bifidobacterium longum* NCC2705. | 17.5 |
|  | *Corynebacterium glutamicum* ATCC 13032 (Bielefeld). | 21.3 |
|  | *Bifidobacterium longum* DJO10A. | 17.0 |
|  | *Mycobacterium tuberculosis* F11. | 22.8 |
|  | *Bifidobacterium adolescentis* ATCC 15703. | 17.5 |
| Firmicutes | | **16.6** |
|  | *Caldicellulosiruptor saccharolyticus* DSM 8903. | 12.1 |
|  | *Clostridium beijerinckii* NCIMB 8052. | 16.9 |
|  | *Bacillus amyloliquefaciens* FZB42. | 20.4 |
|  | *Lactobacillus acidophilus* NCFM. | 19.9 |
|  | *Bacillus cereus* ATCC 10987. | 19.1 |
|  | *Bacillus* subtilis subsp. *subtilis* str. 168. | 19.7 |
|  | *Streptococcus* *pneumoniae* CGSP14. | 14.5 |
|  | *Clostridium* *kluyveri* DSM 555. | 14.5 |
|  | *Lactobacillus* *reuteri* F275. | 14.8 |
|  | *Lactobacillus* *salivarius* UCC118. | 13.7 |
| Proteobacteria | | **19.8** |
|  | *Shewanella* *halifaxensis* HAW-EB4. | 26.7 |
|  | *Campylobacter* *jejuni* subsp. *doylei* 269.97. | 13.7 |
|  | *Helicobacter* *pylori* Shi470. | 18.5 |
|  | *Pseudomonas* *putida* W619. | 23.9 |
|  | *Syntrophobacter* *fumaroxidans* MPOB. | 17.7 |
|  | *Rhizobium* *etli* CIAT 652. | 19.8 |
|  | *Escherichia* *coli* ATCC 8739. | 20.9 |
|  | *Escherichia* *coli* O157-H7 str. Sakai. | 18.6 |
|  | *Actinobacillus* *succinogenes* 130Z. | 20.5 |
|  | *Helicobacter* *acinonychis* str. Sheeba. | 17.4 |
| Bacteroidetes | | **24.9** |
|  | Candidatus *Amoebophilus* *asiaticus* 5a2. | 14.1 |
|  | *Parabacteroides* *distasonis* ATCC 8503. | 28.8 |
|  | *Porphyromonas* *gingivalis* W83. | 18.8 |
|  | *Flavobacterium* *johnsoniae* UW101. | 28.7 |
|  | *Bacteroides* *vulgatus* ATCC 8482. | 28.6 |
|  | *Bacteroides* *fragilis* YCH46. | 28.0 |
|  | *Gramella* *forsetii* KT0803. | 25.2 |
|  | *Salinibacter* *ruber* DSM 13855. | 19.0 |
|  | *Porphyromonas* *gingivalis* ATCC 33277. | 21.8 |
|  | *Bacteroides* *thetaiotaomicron* VPI-5482. | 32.0 |
|  | *Bacteroides* *fragilis* NCTC 9343. | 29.1 |
|  | *Cytophaga* *hutchinsonii* ATCC 33406. | 26.8 |
|  | *Flavobacterium* *psychrophilum* JIP02/86. | 22.5 |
| Chlamydiae | | **15.8** |
|  | *Chlamydia* *trachomatis* D/UW-3/CX | 15.6 |
|  | *Chlamydophila* *felis* Fe/C-56 | 16.7 |
|  | Candidatus *Protochlamydia* amoebophila UWE25 | 12.1 |
|  | *Chlamydophila* *abortus* S26-3 | 15.9 |
|  | *Chlamydophila* *caviae* GPIC | 18.2 |
|  | *Chlamydia* *muridarum* Nigg | 16.3 |
|  | *Chlamydophila* *pneumoniae* J138 | 16.0 |
|  | *Chlamydophila* *pneumoniae* AR39 | 15.5 |
|  | *Chlamydia* *trachomatis* 434/Bu | 15.7 |
|  | *Chlamydia* *trachomatis* L2b/UCH-1/proctitis | 15.7 |
|  | *Chlamydophila* *pneumoniae* CWL029 | 15.9 |
|  | *Chlamydia* *trachomatis* A/HAR-13 | 15.9 |
|  | *Chlamydophila* *pneumoniae* TW-183 | 15.6 |
| Spirochetes | | **17.4** |
|  | *Leptospira* *interrogans* serovar *Copenhageni* str. Fiocruz L1-130 C1 | 16.6 |
|  | *Treponema* *denticola* ATCC 35405 | 20.7 |
|  | *Leptospira* *interrogans* serovar *Lai* str. 56601 C1 | 12.9 |
|  | *Treponema* *pallidum* subsp. *pallidum* str. Nichols | 18.7 |
|  | *Borrelia* *burgdorferi* B31 | 21.6 |
|  | *Leptospira* *biflexa* serovar *Patoc* strain 'Patoc 1 (Ames)' C1 | 22.3 |
|  | *Treponema* *pallidum* subsp. *pallidum* SS14 | 18.7 |
|  | *Borrelia* *hermsii* DAH | 13.6 |
|  | *Leptospira* *biflexa* serovar *Patoc* strain 'Patoc 1 (Paris)' C1 | 19.4 |
|  | *Borrelia* *afzelii* PKo | 15.5 |
|  | *Leptospira* *borgpetersenii* serovar *Hardjo*-*bovis* JB197 C1 | 16.4 |
|  | *Leptospira* *borgpetersenii* serovar *Hardjo*-*bovis* L550 C1 | 16.6 |
|  | *Borrelia* *garinii* PBi | 13.6 |
| Aquificales & Thermotogae | | **13.7** |
|  | *Thermotoga* *maritima* MSB8 | 13.4 |
|  | *Thermotoga* sp. RQ2 | 14.7 |
|  | *Fervidobacterium* *nodosum* Rt17-B1 | 13.6 |
|  | *Thermotoga* *lettingae* TMO | 17.7 |
|  | *Thermosipho* *melanesiensis* BI429 | 12.0 |
|  | *Thermotoga* *petrophila* RKU-1 | 14.1 |
|  | *Petrotoga* *mobilis* SJ95 | 13.0 |
|  | *Aquifex* *aeolicus* VF5 | 11.5 |
|  | *Sulfurihydrogenibium* sp. YO3AOP1 | 13.7 |
| Verrucomicrobiae | | **25.6** |
|  | *Verrucomicrobium* *spinosum* DSM 4136 | 27.2 |
|  | *Opitutus* *terrae* PB90-1 | 30.4 |
|  | *Akkermansia* *muciniphila* ATCC BAA-835 | 25.3 |
|  | *Methylacidiphilum* *infernorum* V4 | 13.1 |
|  | *Chthoniobacter* *flavus* Ellin428 | 29.6 |
|  | *Opitutaceae* *bacterium* TAV2 | 25.1 |
|  | *Pedosphaera* *parvula* Ellin514 | 28.7 |
