# Supplementary material for: The Genome of Akkermansia muciniphila, a Dedicated Intestinal Mucin Degrader, and Its Use in Exploring Intestinal Metagenomes
Source: PLoS One. 2011 Mar 3;6(3):e16876. doi: 10.1371/journal.pone.0016876 (PMC3048395; doi:10.1371/journal.pone.0016876)
Supplement: Table S3 — List of Akkermansia muciniphila protein coding genes that include mononucleotide repeats of 9 bp or longer. The relative gene position (between 0 and 1) is calculated based on the start (relative gene position 0) and end (relative gene position of 1) of each gene. (DOCX) [file pone.0016876.s004.docx]

Supplementary Table S4. The presence of 16S ribosomal sequences (>95% identical to that of *Akkermansia muciniphila*) in the metagenomic databases.

| # | **sample** | **contig** | **length** | **Identity (%)** |
| --- | --- | --- | --- | --- |
| 2 | B | MC20.MG17.AS1.I1.C764 | 1505 | 98.14 |
| 7 | E | MC20.MG20.AS1.I1.C1424 | 1177 | 99.58 |
| 15 | G | MC20.MG21.AS1.I19.R49690 | 482 | 99.59 |
| 22 | MH12 | MC20.MG31.AS1.I1.C2291 | 1505 | 99.93 |
| 23 | MH13 | MC20.MG30.AS1.I1.C1293 | 1505 | 99.87 |
| 26 | NO1 | MC20.MG22.AS1.I18.C49 | 751 | 100 |
| 27 | NO3 | MC20.MG23.AS1.I1.C779 | 1505 | 99.87 |
| 28 | NO4 | MC20.MG24.AS1.I27.R17509 | 614 | 100 |
|  |  | MC20.MG24.AS1.I1.C1569 | 522 | 99.81 |
| 32 | OB6 | MC20.MG28.AS1.I17.R4182 | 639 | 100 |
|  |  | MC20.MG28.AS1.I17.R50689 | 522 | 100 |
|  |  | MC20.MG28.AS1.I17.R50996 | 484 | 100 |
|  |  | MC20.MG28.AS1.I17.R17964 | 420 | 98.33* |
| 33 | OB8 | MC20.MG37.AS1.I24.R3654 | 638 | 99.84 |
|  |  | MC20.MG37.AS1.I24.R1653 | 479 | 98.75* |
| 37 | UC6 | MC20.MG36.AS1.I1.C880 | 1505 | 99.80 |

*) contains ambiguous nucleotides (N), resulting in low identity values; corrected identities are >99%.
