# Supplementary material for: The Genome of Akkermansia muciniphila, a Dedicated Intestinal Mucin Degrader, and Its Use in Exploring Intestinal Metagenomes
Source: PLoS One. 2011 Mar 3;6(3):e16876. doi: 10.1371/journal.pone.0016876 (PMC3048395; doi:10.1371/journal.pone.0016876)
Supplement: Table S6 — General characteristics of the 37 metagenomes used in this study (metagenomes that contain predicted Akkermansia sequences are indicated in green). (DOCX) [file pone.0016876.s007.docx]

Supplementary Table S6. General characteristics of the 37 metagenomes used in this study (metagenomes that contain predicted *Akkermansia* sequences are indicated with a *).

| # | **Title** | **Project** | **Source^** | **Sample** | **Nationality** | **Gender** | **Age** | **Physiological_status** | **Counts** | **Total size** | **Amount of Akkermansia DNA (>200 bp, >90% identity)** | **Number of contigs** | **Relative abundance (%)** | **HitChip relative abundance (%)** | **Number of 16S rRNA hits (% identity)** |
| --- | --- | --- | --- | --- | --- | --- | --- | --- | --- | --- | --- | --- | --- | --- | --- |
| 1* | IT-AD-1 | microage |  | A | italian | F | 84 | elderly | 57133 | 49201030 | 211602 | 275 | 0.43 |  |  |
| 2* | IT-AD-2 | microage |  | B | italian | M | 87 | elderly | 59564 | 53016574 | 155799 | 107 | 0.29 |  | 1 (98.14) |
| 3* | IT-AD-3 | microage |  | C | italian | F | 77 | elderly | 73824 | 61489938 | 310164 | 428 | 0.5 |  |  |
| 4 | ES-AD-1 | metahit | Project ID: 33059 | CD1 | spanish | F | 25 | CD | 65088 | 63576076 | 0 |  |  | 0.017 |  |
| 5* | ES-AD-2 | metahit | Project ID: 33061 | CD2 | spanish | M | 49 | healthy relative of CD1 | 85009 | 70601731 | 17168 | 25 | 0.02 | 0.015 |  |
| 6 | IT-AD-4 | microage |  | D | italian | M | 80 | elderly | 38297 | 36284293 | 0 |  |  |  |  |
| 7* | IT-AD-5 | microage |  | E | italian | M | 70 | elderly | 77082 | 63135227 | 1384051 | 1410 | 2.19 |  | 1 (99.58%) |
| 8* | JP-AD-1 | kurokawa07 | BAAU01000001-BAAU01028900 | F1-S | japanese | M | 30 |  | 31096 | 35575967 | 22174 | 24 | 0.06 |  |  |
| 9 | JP-AD-2 | kurokawa07 | BAAV01000001-BAAV01036326 | F1-T | japanese | F | 28 |  | 37749 | 39020190 | 0 |  |  |  |  |
| 10 | JP-IN-1 | kurokawa07 | BAAW01000001-BAAW01016539 | F1-U | japanese | F | 0.58 |  | 17588 | 22815676 | 0 |  |  |  |  |
| 11* | JP-AD-3 | kurokawa07 | BAAX01000001-BAAX01036455 | F2-V | japanese | M | 37 |  | 37793 | 41313262 | 35594 | 39 | 0.09 |  |  |
| 12* | JP-AD-4 | kurokawa07 | BAAY01000001-BAAY01030198 | F2-W | japanese | F | 36 |  | 31171 | 36156855 | 6826 | 9 | 0.02 |  |  |
| 13* | JP-AD-5 | kurokawa07 | BAAZ01000001-BAAZ01031237 | F2-X | japanese | M | 3 |  | 31685 | 34467680 | 309012 | 327 | 0.9 |  |  |
| 14* | JP-AD-6 | kurokawa07 | BABA01000001-BABA01035177 | F2-Y | japanese | F | 1.5 |  | 36803 | 41718743 | 54428 | 58 | 0.13 |  |  |
| 15* | IT-AD-6 | microage |  | G | italian | F | 72 | elderly | 75435 | 62138255 | 195493 | 267 | 0.31 |  | 1 (99.59%) |
| 16 | JP-AD-7 | kurokawa07 | BABB01000001-BABB01020226 | In-A | japanese | M | 45 |  | 21092 | 24491884 | 0 |  |  |  |  |
| 17 | JP-AD-8 | kurokawa07 | BABC01000001-BABC01009958 | In-B | japanese | M | 0.5 |  | 6791 | 10687920 | 0 |  |  |  |  |
| 18 | JP-AD-9 | kurokawa07 | BABD01000001-BABD01037296 | In-D | japanese | M | 35 |  | 38642 | 39888261 | 0 |  |  |  |  |
| 19 | JP-IN-2 | kurokawa07 | BABE01000001-BABE01020532 | In-E | japanese | M | 0.25 |  | 15971 | 19473697 | 0 |  |  |  |  |
| 20* | JP-IN-3 | kurokawa07 | BABF01000001-BABF01016164 | In-M | japanese | F | 0.33 |  | 17802 | 23882918 | 575 | 1 | 0 |  |  |
| 21 | JP-IN-4 | kurokawa07 | BABG01000001-BABG01034797 | In-R | japanese | F | 24 |  | 34389 | 38225044 | 0 |  |  |  |  |
| 22* | DA-AD-3 | metahit | Project ID: 33055 | MH12 | danish | F | 49 | obese | 110201 | 93505669 | 1881572 | 1560 | 2.01 | 0.055 | 1 (99.93%) |
| 23* | DA-AD-2 | metahit | Project ID: 33053 | MH13 | danish | M | 54 | healthy | 99166 | 83354756 | 2538185 | 1102 | 3.05 | 0.127 | 1 (99.87%) |
| 24 | DA-AD-4 | metahit | Project ID: 33057 | MH30 | danish | M | 59 | obese | 113540 | 96152661 | 0 |  |  | 0.032 |  |
| 25* | DA-AD-1 | metahit | Project ID: 33049 | MH6 | danish | F | 59 | healthy | 105516 | 86753488 | 10407 | 16 | 0.01 | 0.017 |  |
| 26* | FR-AD-1 | microobes | (Project ID: 33305 | NO1 | french | M | 63 | healthy | 81876 | 67366915 | 524000 | 678 | 0.78 |  | 1 (100%) |
| 27* | FR-AD-2 | microobes | Project ID: 33307 | NO3 | french | M | 61 | healthy | 73590 | 57125037 | 2253202 | 1398 | 3.94 |  | 1 (99.87%) |
| 28* | FR-AD-3 | microobes | Project ID: 33309 | NO4 | french | M | 60 | healthy | 70331 | 57189273 | 1577353 | 1565 | 2.76 |  | 2 (100%, 99.81%) |
| 29* | FR-AD-4 | microobes | Project ID: 33311 | NO8 | french | M | 60 | healthy | 87546 | 67187941 | 620695 | 793 | 0.92 |  |  |
| 30 | FR-AD-5 | microobes | Project ID: 38231 | OB1 | french | M | 64 | obese | 78155 | 59651934 | 0 |  |  |  |  |
| 31 | FR-AD-6 | microobes | Project ID: 33313 | OB2 | french | M | 63 | obese | 83931 | 66168175 | 0 |  |  |  |  |
| 32* | FR-AD-7 | microobes | Project ID: 38233 | OB6 | french | M | 62 | obese | 71160 | 57081404 | 585486 | 796 | 1.03 |  | 4 (100%, 100%, 100%, 98.33%) |
| 33* | FR-AD-8 | microobes | Project ID: 45929 | OB8 | french | M | 60 | obese | 77225 | 57168299 | 942798 | 1201 | 1.65 |  | 2 (99.84%, 98.75%) |
| 34 | AM-AD-1 | gill06 | AAQK00000000 | Subject7 | american | F | 28 | healthy | 41831 | 46136049 | 0 |  |  |  |  |
| 35 | AM-AD-2 | gill06 | AAQL00000000 | Subject8 | american | M | 37 | healthy | 37448 | 46003405 | 0 |  |  |  |  |
| 36* | ES-AD-3 | metahit | Project ID: 33113 | UC4 | spanish | F | 47 | UC | 98656 | 79886227 | 747 | 1 | 0 | 0.017 |  |
| 37* | ES-AD-4 | metahit | Project ID: 33063 | UC6 | spanish | F | 38 | healthy | 115558 | 94544463 | 2294676 | 1409 | 2.43 | 0.017 | 1 (99.80%) |
|  |  |  |  |  |  |  |  |  |  |  |  |  |  |  |  |
|  |  |  |  |  |  |  |  |  |  | **Total** | **15,932,007** | **13,489** |  |  |  |

^)Sources are genome project IDs or accession numbers from the GenBank databases.
