# Supplementary material for: The Genome of Akkermansia muciniphila, a Dedicated Intestinal Mucin Degrader, and Its Use in Exploring Intestinal Metagenomes
Source: PLoS One. 2011 Mar 3;6(3):e16876. doi: 10.1371/journal.pone.0016876 (PMC3048395; doi:10.1371/journal.pone.0016876)
Supplement: Table S7 — Overview of Akkermansia -like sequences (>95% identity and <98% identity compared to the sequenced A. muciniphila 16S sequence) in 9773 nearly full-length 16S rRNA sequences from a microbiome study in twins [6] . (DOCX) [file pone.0016876.s008.docx]

Supplementary Table S7. Overview of *Akkermansia*-like sequences (>95% identity and <98% identity compared to the sequenced *A. muciniphila* 16S sequence) in 9773 nearly full-length 16S rRNA sequences from a microbiome study in twins [6].

| **Individual** | **Number of 16S rRNA sequences** | **Number of hits (>95%, >500 bp)** | **Number of hits (<98% identity)** |
| --- | --- | --- | --- |
| TS1 | 341 | 23 | 2 |
| TS2 | 350 | 19 |  |
| TS3 | 326 |  |  |
| TS4 | 341 |  |  |
| TS5 | 342 |  |  |
| TS6 | 334 | 19 | 2 |
| TS7 | 236 | 1 |  |
| TS8 | 354 | 5 |  |
| TS9 | 356 | 3 |  |
| TS13 | 331 |  |  |
| TS14 | 342 |  |  |
| TS15 | 335 |  |  |
| TS19 | 330 |  |  |
| TS20 | 335 | 2 |  |
| TS21 | 330 | 7 |  |
| TS25 | 350 | 1 |  |
| TS26 | 247 | 3 |  |
| TS27 | 340 | 6 |  |
| TS28 | 341 |  |  |
| TS29 | 335 | 5 |  |
| TS30 | 254 |  |  |
| TS49 | 328 |  |  |
| TS50 | 316 |  |  |
| TS51 | 325 | 70 | 5 |
| TS55 | 348 |  |  |
| TS56 | 278 |  |  |
| TS57 | 335 |  |  |
| TS148 | 308 | 2 | 2 |
| TS149 | 337 |  |  |
| TS150 | 348 | 25 | 1 |
| **Total** | **9773** | **192** | **12** |
